# Tuberculosis care for pregnant women: a systematic review

**DOI:** 10.1186/s12879-014-0617-x

**Published:** 2014-11-19

**Authors:** Hang Thanh Nguyen, Chiara Pandolfini, Peter Chiodini, Maurizio Bonati

**Affiliations:** Department of Public Health, Laboratory of Maternal and Child Health, IRCCS - Istituto di Ricerche Farmacologiche Mario Negri, Via G. La Masa 19, Milan, Italy; Department of Clinical Parasitology, University College London Hospitals NHS Foundation Trust, London, UK

**Keywords:** Delivery of health care, Pregnancy, Tuberculosis, Women

## Abstract

**Background:**

Tuberculosis (TB) during pregnancy may lead to severe consequences affecting both mother and child. Prenatal care could be a very good opportunity for TB care, especially for women who have limited access to health services. The aim of this review was to gather and evaluate studies on TB care for pregnant women.

**Methods:**

We used a combination of the terms “tuberculosis” and “pregnancy”, limited to human, to search for published articles. Studies reflecting original data and focusing on TB care for pregnant women were included. All references retrieved were collected using the Reference Manager software (Version 11).

**Results:**

Thirty five studies were selected for review and their data showed that diagnosis was often delayed because TB symptoms during pregnancy were not typical. TB prophylaxis and anti-TB therapy appeared to be safe and effective for pregnant women and their babies when suitable follow up and early initiation were present, but the compliance rate to TB prophylaxis is still low due to lack of follow up and referral services. TB care practices in the reviewed studies were in line in principle with the WHO International Standards for Tuberculosis Care (ISTC).

**Conclusions:**

Integration of TB care within prenatal care would improve TB diagnosis and treatment for pregnant women. To improve the quality of TB care, it is necessary to develop national level guidelines based on the ISTC with detailed guidelines for pregnant women.

**Electronic supplementary material:**

The online version of this article (doi:10.1186/s12879-014-0617-x) contains supplementary material, which is available to authorized users.

## Background

According to the World Health Organization (WHO), every year about 700,000 women die of tuberculosis (TB) and over three million contract the disease [1]. TB is the third leading cause of death among women aged 15–44. TB can cause infertility and contributes to poor reproductive health outcomes [2],[3].

When pregnant women contract TB, the disease is more difficult to diagnose because TB symptoms such as fatigue, shortness of breath, sweating, tiredness, cough, and mild fever are similar to physiological symptoms of pregnancy. Untreated TB or TB treated late may lead to severe consequences affecting both mother and child [4],[5]. Pregnant women with pulmonary TB who are treated appropriately do not have increased rates of maternal or neonatal complications, while without treatment, TB can lead to increased neonatal morbidity, low birth weight, prematurity, and increased pregnancy complications, including four-fold increases in maternal morbidity due to higher rates of abortion, post partum haemorrhage, labour difficulties, and pre-eclampsia [5]. Prenatal care could be a very good opportunity for TB screening and diagnosis and for following up TB care, especially for women who have limited access to health services, such as migrants or women of limited social/economic status, who only approach medical services when pregnant [4],[5].

The WHO Guidelines for Treatment of Tuberculosis provide recommendations for TB care and recommend the integration of TB care within both prenatal care procedures and the Preventing Mother to Child Transmission of HIV Program (PMTCT) in order to utilise existing health resources and systems to improve accessibility and effectiveness of TB care for pregnant women and prevent the mother to child transmission of TB [6].

Regardless of the importance of TB care in the prenatal period, however, only a modest number of articles addressing TB in pregnant women have been published. Some major controversial issues in TB care during pregnancy remain that require further research, such as the safety, reliability, and feasibility of TB screening methods used in the prenatal period [7], drug therapy for pregnant MDR women [8], and delay of treatment until the post partum period in case of latent tuberculosis infection (LTBI) [9]. LTBI is a condition in which a person is infected with Mycobacterium tuberculosis, but does not currently have active tuberculosis disease.

This systematic review aimed to gather and evaluate evidence based studies on TB care in pregnancy, with consideration of WHO standard guidelines for TB care, in order to recommend better practices to improve TB care for pregnant women. For studies on TB care in pregnancy the authors intended those addressing screening/diagnosis, prevention, treatment, or follow-up/supervision/counselling activities/services for women in pregnancy.

## Methods

The following databases were searched for articles in English, published in any year up to December 31, 2012: MEDLINE (indexes articles dating back to 1946), EMBASE (1973), the Cochrane Library (various), and www.clinicaltrials.gov (2000). The search strategies involved 1. using the MeSH terms “tuberculosis” and “pregnancy”, limited to human; 2. the terms “tuberc*” and “pregnan*” as free text within articles indexed in the last 90 days, limited to human; 3. combining the results of MeSH “tuberculosis” and free text “tuberc*”; 4. combining the results of MeSH “pregnancy” and free text “pregnan*”; 5. combining the results of steps 3 and 4 (pregnancy AND tuberculosis, limited to Human). The syntax was adjusted for the specific databases. Reference lists were then searched for potentially relevant articles.

### Inclusion criteria

Studies reflecting original data and focusing on TB care (screening, diagnosis, treatment, and follow up) in pregnant women.

### Exclusion criteria

Articles were excluded if: 1. The target group was NOT pregnant women, 2. TB was mentioned, but NOT TB care (i.e. TB as a complication of other diseases, as part of a study on infectious diseases, etc.); 3. Original data was not included 4. They were NOT studies (letters, presentations, conference documents, case reports).

**Figure 1:**
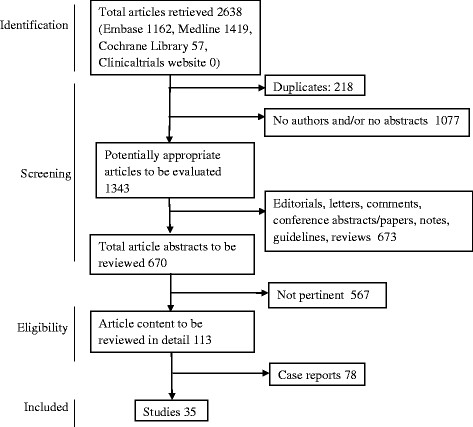
**PRISMA Flow diagram of search strategy.**

Figure 1 summarizes the selection process for review articles with detailed number of articles in each step.

### Data extraction and assessment

All references retrieved were collected and analysed using the Reference Manager (Version 11) program. All identified abstracts were read for their applicability to inclusion and exclusion criteria by two co-reviewers and discrepancies were resolved by a third reviewer. Potentially relevant articles were obtained and examined, and the quality of selected studies was assessed by two evaluators using the checklists from the National Institute for Health and Clinical Excellence (NICE)’s manual for developing public health guidance [10] (See Additional file 1). Each study was evaluated by the appropriate NICE checklist depends on study type. The quality evaluation took into consideration the studies’ internal and external validity. According to NICE guidelines, a study was rated good quality (++) if it fulfilled all or most of criteria in the check list, medium quality if it fulfilled some (+), and low quality if few or no criteria were fulfilled (−) [10]. Inter-reviewer reliability was measured using Cohen’s Kappa statistics. Discrepancies were addressed by a third reviewer. MS Excel software was used to process data from the selected studies.

### Details of ethics approval

This is a systematic review of previously published data and therefore does not require ethical approval.

## Results

### Summary of included studies

Thirty five studies were selected for detailed review. Good inter-rater agreement on the quality assessment of the studies was found (K = 0.70). There were 6 studies with good quality (++), 21 with medium quality (+), and 8 with low quality (−). The reasons to include studies with low quality is the limited number of studies in selected topics and one of the purpose of this review is to have an overall evaluation on the situation of research on TB care for pregnant women.

Table 1 shows the characteristics of the reviewed studies and the quality evaluation scores. Concerning the study types, there were 14 cohort studies, 8 before and after studies, 6 cross sectional studies, 4 case control studies, 2 controlled trials, and 1 qualitative study.

**Table 1:** **Characteristics of reviewed studies and quality evaluation results, sorted by quality evaluation score**

No.	Authors/Year published (reference)	Year data collected	Study type	Topic	No. of cases	Length of study	Country	Quality score* (from high to low)
1	Worjoloh et al./2011 [11]	5/2009 – 3/2010	Cross sectional	Screening	220	10 months	US	++
2	Sangala et al./2006 [12]	2006	Qualitative	Screening	40 (15 pregnant, 15 non pregnant, 10 antenatal care providers)	N/A	Malawi	++
3	Gounder et al./2011 [13]	12/2008-7/2009	Cross sectional	Screening, prevention	3963	7 months	South Africa	++
4	Czeizel et al./2001 [14]	1980-1996	Case control	Treatment	61016 (38151 controls and 22865 cases)	16 years	Hungary	++
5	Tripathy et al./2003 [15]	1986-2001	Prospective cohort study	Treatment	213 (111 pregnant with TB, 51 pregnant without TB, 51 non pregnant with TB)	15 years	India	++
6	Jana et al./1999 [16]	1983-1993	Case control	Treatment	165 (33 pregnant with TB, 132 pregnant without TB)	10 years	India	++
7	Figueroa-Damian et al./1998 [17]	1990-1995	Case control	Treatment	100 (25 pregnant with TB, 75 pregnant without TB)	5 years	Mexico	+
8	Carter et al. /1994 [18]	1987-1991	Retrospective cohort study	Diagnosis	22 (7 pregnant, 15 nonpregnant)	4 years	US	+
9	Doveren et al./1998 [19]	1990-1996	Retrospective cohort study	Diagnosis	14 (5 pregnant, 9 non pregnant)	6 years	Netherlands	+
10	Present et al./1975 [20]	1975	Non-randomized controlled trial	Diagnosis	326 (167 pregnant, 226 non- pregnant)	1 year	US	+
11	Jonnalagadda et al./2010 [21]	1997 - 2005	Prospective cohort study	Diagnosis	333	8 years	Kenya	+
12	Khan et al./2001 [22]	1996-1998	Prospective study cohort 1997–1998, retrospective cohort study 1996	Diagnosis	101	3 years	South Africa	+
13	Knight et al./2009 [23]	2005-2006	Cross sectional	Diagnosis	33	1 year	UK	+
14	Kothari et al./2006 [24]	1/1997-12/2001	Before and after study	Diagnosis, treatment, follow-up	32	5 year	UK	+
15	Kwara et al./2008 [25]	2003	Retrospective cohort study	Follow up	845 (97 pregnant women)	1 year	US	+
16	Cruz et al./2005 [26]	2000	Retrospective cohort study	Follow-up	425	1 year	US	+
17	Kali et al./2006 [27]	6/2003-10/2003	Cross sectional	Screening	370	4 months	South Africa	+
18	Sepulveda et al./1995 [28]	1991-1994	Prospective cohort study	Screening	840	3 years	Chile	+
19	Meints et al./2010 [29]	2003-2006	Cross sectional	Screening	1767	4 years	US	+
20	Sheriff et al./2010 [30]	6/2008-8/2008)	Cross sectional	Screening	286	2 months	Tanzania	+
21	Mofenson et al./1995 [31]	9/1989 -3/1993	Prospective cohort study	Screening, diagnosis	183 (65 pregnant and 118 non-pregnant)	3.5 years	US and Puerto Rico	+
22	Pillay et al./2001 [32]	1996-1998	Prospective cohort study	Screening, diagnosis	146	2 years	South Africa	+
23	Margono et al./1994 [33]	1985-1992	Before and after study	Screening, diagnosis, treatment	16	7 years	US	+
24	Lighter-Fisher et al./2012 [34]	2012	Non-randomized controlled trial	Screening, diagnosis	280 (140 pregnant women, 140 non pregnant women)	N/A	US	+
25	Donald et al./1991 [35]	1991	Retrospective cohort study	Treatment	30 children with mothers who received streptomycin injection during pregnancy	N/A	South Africa	+
26	Palacios et al./2009 [36]	1996 -2005	Before and after study	Treatment (drug resistant)	38	10 years	Peru	+
27	Tabarsi et al./2011 [37]	2003-2009	Before and after study	Treatment (drug resistant)	5	6 years	Iran	+
28	Cheng et al./2003 [38]	2002	Before and after study	Diagnosis, treatment	29	Done in 2002 with article search 1966-2002	Hong Kong, China	-
29	Franks et al./1989 [39]	1980-1982	Case control	Prevention	7629 (3681 pregnant, 3948 non-pregnant)	18 months	US	-
30	Sackoff et al./2006 [40]	1999-2000	Retrospective cohort study	Prevention, follow-up	730	1 year	US	-
31	Gupta et al./2011 [41]	2002-2007	Retrospective cohort study	Screening	799	5 years	India	-
32	Keskin et al./2008 [42]	2000-2005	Before and after study	Treatment	5	5 years	Turkey	-
33	De Oliveira et al./2011 [43]	1995-2007	Before and after study	Treatment (drug resistant)	7	13 years	Brazil	-
34	Khan et al./2007 [44]	1996-2001	Before and after study	Treatment (drug resistant)	5	5 years	South Africa	-
35	Llewelyn et al./2000 [45]	12/1995-5/1998	Prospective cohort study	Diagnosis, treatment	13	30 months	UK	-

*(++): High quality; (+) Medium quality; (−) Low quality.

Concerning the topics addressed, some studies addressed multiple topics: 8 studies covered 2 topics and 1 study covered 3. The topic of TB diagnosis/screening was presented in 21 studies, 14 studies addressed TB treatment (including 4 on MDR treatment), 2 studies were on TB prevention, specifically on LTBI prophylaxis, and 3 studies were on the follow-up of women’s compliance to the TB prophylaxis therapy. All studies were published after 1975, with a majority (26/35) published after 1999. Data originated from 16 different countries. Fifteen studies were carried out in resource-rich countries: US (11 studies), UK (3), and the Netherlands (1), while 20 were carried out in resource-limited countries: South Africa (6 studies), India (3), and the remaining countries had 1 each. One group of researchers in South Africa conducted 3 studies [22],[32],[44], other studies were all conducted by different groups of researchers. In all, 81093 people were enrolled in these studies, 37404 of whom in the study group (pregnant women with TB) and 43689 in the controlled/comparison groups (pregnant women without TB or/and non-pregnant women with TB). The large total was mostly due to one population based study involving 61016 people [14].

### TB diagnosis and screening for pregnant women

The procedures for TB screening and diagnosis for pregnant women described in the reviewed studies include the tuberculin sensitivity test (TST/PPD) followed by the sputum test (Acid-fast bacillus - AFB) and the shielded chest X-ray [18],[19],[23],[24],[30]. The AFB smear test appears to have low sensitivity in pregnant women [18],[19],[30], but is still used in low resource settings as part of the procedure for diagnosing active TB due to its low cost and simple technique [13],[30]. AFB culture was used as a confirmation of diagnosis, but is time consuming and not available in low resource settings [13],[23]. Another technique, fluorescence microscopy, was recommended as a substitute for AFB culture because it is cheaper [13]. This procedure is for pulmonary TB cases and cannot identify extra pulmonary TB without additional tests and presence of physical TB symptoms. A clinical examination comprising a questionnaire tracking TB history and detecting TB clinical symptoms was also used and was proven to increase reliability of TB screening and diagnosis when combined with paraclinical tests [13],[23],[27],[30],[41]. Some authors recommended not using chest X-ray for pregnant women if there were no clinical symptoms of TB [30].

TST is used widely as the first step in TB screening and diagnosis and to identify LTBI [18]. Studies showed that pregnancy does not affect the sensitivity of this test [20], but its result can be affected by HIV infection or any situation that severely weakens the immune system (such as disseminated TB), as these could lead to false negative results [21],[30],[45]. BCG vaccination can also lead to TST positive results in healthy women [11],[28]. In a high HIV prevalence setting, other tests and clinical symptoms should therefore be taken into account in diagnosing TB [41] and the TST and anergy skin tests (the latter is used to evaluate whether the immune system is functioning properly or not and can indicate whether the results of the other skin test are reliable) are recommended as a TB screening method in the prenatal care procedures [31]. In populations in which the majority of people are BCG vaccinated or their vaccination status is uncertain, TST is discouraged and IGRA is recommended for TB screening and diagnosis [11],[28].

Concerning the IGRA test, one study in Kenya compared results of this test with the TST in screening for TB and showed the advantage of the IGRA test over TST in TB screening and diagnosis for HIV positive pregnant women, since its sensitivity is not affected by HIV infection [21]. Two studies proved the value of IGRA in detecting LTBI in pregnant women, since the results of this test were not affected by BCG vaccination, thus avoiding the TST false positive result and the unnecessary, consequent INH prophylaxis [11],[21].

### Prevention

TB prevention includes BCG vaccination in childhood and INH prophylaxis for LTBI positive people. There were 2 studies on TB prevention and both were on INH prophylaxis for LTBI pregnant women. Both were conducted in the US with pregnant women of foreign origin and LTBI was diagnosed by TST [39],[40]. One study [40] showed a low completion rate of INH therapy ( 9.3% ) and the other showed a high risk of INH toxic hepatitis, with pregnant women having a 2.5 fold greater risk of INH hepatitis than non-pregnant women (but this result was not statistically significant due to the small number of women) [39]. The 2 studies found that the main reason for this discouraging result was a lack of follow up and referral services for pregnant women undergoing INH prophylaxis [39],[40].

### Treatment

There were 375 pregnant women with TB in the 14 studies on TB treatment. Treatment outcome was generally positive, with 332/375 women cured (confirmed by AFB culture conversion). In terms of mortality, 25 women died during treatment, 11 of whom died due to meningitis TB, 11 due to MDR-TB, 2 due to acute respiratory distress syndrome (ARDS), and 1 due to a non-TB related reason (massive pulmonary embolism). Other than mortality, the negative treatment outcomes included 4 treatment failures, 4 cases of residual functional deficit, 7 treatment terminations due to adverse drug effects, and 3 cases of treatment abandonment.

In terms of pregnancy outcome, only 11 women chose to terminate the pregnancy when they discovered their TB situation, while the others continued the pregnancy and underwent TB therapy. Among the pregnant women undergoing TB therapy, 332 women gave birth, 1 had a therapeutic abortion, 3 had miscarriages, 3 had stillborns, and 25 died. Concerning the 332 cases of mothers who gave birth, 4 infants died shortly after birth due to pneumonia and prematurity, 2 were HIV positive, 1 had active TB, 2 had LTBI, 50 were low birth weight, and 7 had growth restriction. Studies also showed that HIV infected women were more likely to choose pregnancy termination and had higher mortality and morbidity rates, even with intensive TB treatment combined with HIV treatment [33],[44].

The first line drugs used for pregnant women in the studies included INH, ethambutol (ETB), rifampicin (RIF), and, in some cases of extra pulmonary TB, pyrazinamid (PZA) [15],[19],[33],[45]. In the MDR cases, the second line drugs, including drugs of the amino glycosides group, fluoroquinolone, thioamides, cycloserines, and terizidone, were used in combination with effective first line drugs, and the treatment regime depended on the drug resistance situation of the individual cases [36],[37],[43],[44].

Regarding effectiveness and safety of anti-TB drugs, results of the reviewed studies showed no significant association between child abnormality and mother’s exposure to anti-TB drugs, both for 1^st^ and 2^nd^ line anti-TB drugs during pregnancy [14],[15],[37],[43],[44]. Other significant adverse effects were recorded in a very small number of pregnant women (2 cases of drug induced hepatitis, 2 of PZA allergy, 2 of sensorineural deafness, and 1 of severe nausea and jaundice) and led to termination of therapy without mortality [24],[44]. Streptomycin (SM) was not used in any studies because of its potential risk of deafness in babies. However, a retrospective study was conducted, checking the hearing capacity of 30 children whose mothers received SM injection during pregnancy, and found no significant effect, with only one case of deafness possibly linked to the mother’s use of SM. Authors of this study recommend only using SM after the 2^nd^ trimester if really necessary [35].

There were 4 studies with 55 pregnant women on MDR treatment. Unlike other studies, in these TB was detected in all the women before pregnancy. More specifically, 48/55 of the women had been diagnosed with MDR TB and had already taken 2^nd^ line anti-TB drugs before getting pregnant, while 7/55 were diagnosed MDR TB and started therapy during pregnancy [36],[37],[43],[44]. After being counselled by clinicians, only 6/55 women chose abortion, while the rest decided to continue the pregnancy and undergo MDR therapy [37],[44]. Eleven women died (8 died during treatment and 3 died after completing treatment for unknown reasons). There was one stillbirth and one child died prematurely due to pneumonia. One woman and her child were lost to follow up. One woman had to terminate treatment due to hepatitis. Other cases were treated successfully. The results of the studies showed that, with an attentive follow-up and appropriate therapy, MDR-TB pregnant women can be cured and have a positive maternal outcome, and should therefore be given the option to continue with a pregnancy [36],[37],[44]. The results also showed that a delay in, or default, MDR treatment were the main causes of mortality and morbidity for mothers and babies [36],[43],[44].

### Follow-up

Follow-up actions for TB therapy include checking for a woman’s drug consumption, clinical symptoms of anti-TB drug adverse effects, and liver function tests. Good compliance with TB treatment in pregnant women led to better maternal outcome and TB recovery rate. These studies showed that adequate health services and directly observed therapy (DOT) could greatly contribute to women’s compliance, and, therefore, to treatment success [36],[45].

There were 3 studies on follow-up of TB therapy for pregnant women and all were on INH prophylaxis. Pregnant women with LTBI were offered 6 months of prophylaxis with INH. The compliance rate was low, possibly due to the women’s concern about hepatitis and other adverse effects [25],[40], and the lack of referral services for treatment evaluation and action from health care providers to ensure compliance [40]. Compared to the general population, pregnant women were less likely to initiate the INH prophylaxis – 52.1% vs 14.7% [25]. In all 3 studies, the completion rate among pregnant women was very low (14.7%, 9.3%, and 21.2%) [25],[26],[40]. All studies recommended that health care providers implement better follow-up strategies to increase patient compliance in the prenatal and post-partum periods, ensure follow-up of drug adverse effects, and not dispense INH quantities covering more than 30 days of treatment at each visit.

## Discussion

### Main findings

In resource-rich countries, pregnant women with high TB prevalence are migrants and people of foreign origin [11],[18],[23],[24],[28],[45], while in resource-limited countries, HIV infected pregnant women are the group with high TB prevalence and mortality [13],[22],[27],[32],[41].

The major problem concerning TB diagnosis for pregnant women is the delay in diagnosis, with a median delay time, defined as the duration from symptom onset to confirmation of diagnosis, ranging from 7 days to 6 months. The main reasons for this delay are that women seek health services and prenatal care at a late stage of their pregnancy and that TB during pregnancy is asymptomatic or has nonspecific symptoms, especially in cases of extra pulmonary TB [18],[23],[38],[45]. Compared to non-pregnant women, pregnant women were more likely to be diagnosed with TB via routine screening (as part of prenatal care) [30]. In HIV infected people, the difficulty in diagnosis is even greater since the weak immune reaction may cause false negative TST and make early TB symptoms unclear [21],[32]. Thus, all studies recommended the integration of TB screening in prenatal care procedures for high risk groups.

In terms of treatment, studies have shown the importance of starting treatment as soon as possible, even before TB culture results, to revert the negative impact of TB on mothers and babies [17],[24],[33],[45]. Early treatment of TB (1^st^ and 2^nd^ trimester) led to a maternal outcome comparable to that of non-TB infected pregnant women, and to a much better outcome than that of women who received late treatment [17]. Authors also recommended making TB culture mandatory since it is the most reliable standard for confirming TB infection and treatment effectiveness, and for revising the therapy in case of lack of success [15],[33]. Treatment of extra PTB had a less positive prognosis than PTB due to greater difficulty in diagnosis and treatment [16],[38]. If women are diagnosed and treated with anti-TB early, however, the maternal outcome can be positive [16],[24],[33],[45].

The low LTBI treatment completion rates raised concerns about LTBI prophylaxis during pregnancy. Follow up actions for LTBI in reviewed studies required women go to the health clinics for check-ups and to obtain more medication, but no onsite enforcement in the community/family, such as DOT or visits, was provided to reinforce patient compliance [25],[26],[40]. These studies showed the importance of health services in follow-up, since pregnant women who receive health care from the same clinician before and after delivery, who have insurance, or who receive continuous care from clinics outside hospitals were more likely to complete the therapy [25],[26].

### Strengths and limitations

The strength of the review is that all aspects of TB care in different settings were considered and reviewed and that the authors could therefore use the WHO ISTC as a standard to which to compare TB care practices. However, the review has some limitations. The studies reviewed were of limited quality and covered multiple aspects of TB care, no intensive analysis for each aspect could therefore be performed. There was a limited number of population-based studies, since most were conducted in a single clinic. Results are therefore not solid enough to be applicable to a larger population. Another limitation was that few studies had control groups; some used comparison groups that were not fully comparable with the study group [16]-[18],[20],[25],[28],[31],[39]. Furthermore, the study designs were weak, since no randomised controlled trials were present. Only one qualitative study was found, and with a disease related to socio-economic status and poverty as is TB, additional qualitative studies would have been useful in identifying attitudes, behaviours, challenges, and opportunities for shaping effective interventions/policies for better care for TB patients. A more general limitation is that the differing resources available between different countries make it difficult to generalise the conclusions.

### Interpretation

According to the WHO guidelines, the ISTC applies to the general population, with only a notion on avoiding using streptomycin in treatment during pregnancy [6],[46]. In principle, TB care practices in the reviewed studies were consistent with the ISTC. However, in resource-limited countries some standards could not be put into practice (See Additional file 2).

Since the WHO guidelines focus on resource-limited countries with high TB prevalence, these may not be entirely appropriate for resource-rich countries. The comparison with the ISTC also revealed that, although TB diagnosis and treatment facilities in resource-rich countries are better, the role of counselling and support/supervision has not drawn enough attention on the part of health care providers and researchers in such countries, albeit WHO considers these as important standards to ensure patient compliance to TB treatment.

## Conclusions

Review results have proven both the importance of TB care in reducing TB mortality and morbidity for women and their babies, and the feasibility of TB control interventions, even in limited resource settings. Several recommendations to improve the quality of TB care for pregnant women can be made based on the results of the review:

TB care for pregnant women should utilise available health system resources, especially the antenatal care programs, and should include the patient-centred approach in counselling, supervision, and support as well as a well-managed, nation-wide method of treatment record keeping to ensure patients’ compliance to TB treatment.

Concerning the target of TB care, in resource-rich countries screening interventions should focus on the foreign origin population, while in resource-limited countries interventions should focus on areas with low socio-economic status and high prevalence of HIV infection.

Raising doctors’ awareness on TB is fundamental. When visiting women with unclear symptoms such as fever, doctors should consider TB and investigate the woman’s history and prescribe TB tests in order not to delay diagnosis and to avoid severe consequences.

Concerning TB diagnostic tests, considering the low sensitivity of the AFB smear test in diagnosis for pregnant women and the advantage of the IGRA test over the AFB smear test, IGRA is recommended in diagnosis and screening if possible. Further studies are therefore needed on its specificity and reliability, and on its applicability to a wider population.

Additional studies on TB therapies for pregnant women should be performed, given their scarcity, especially for MDR TB.

Before deciding to start the TB preventive therapy, BCG vaccination status should be confirmed, and during therapy, the test to detect INH adverse effects should be conducted regularly. More active involvement of health care providers in following up women’s compliance could improve the low completion rate of therapy.

Information on individual/family history of TB infection, BCG vaccination, and past treatment, for example, were hardly collected. Collecting such information from patients during the first visit and giving this step high priority could help to improve diagnosis and treatment.

Additional studies, both qualitative and quantitative, and clinical and community-based, need to be performed and should not only address the quality of TB care provided by service providers, but also the behaviours and attitudes of individuals and communities in approaching and using available health services and the barriers faced in accessing and complying with TB treatment. It would be especially important to address issues such as carrying out TB cultures, for which patients then have to return to the clinic for the results, and following up for INH toxicity, since in poor resource settings travelling, for example, is a significant barrier for patients. National TB care guidelines based on the ISTC with detailed guidelines for TB care for pregnant women are necessary. Improvement in TB care for pregnant women will contribute significantly to achieving the Millennium Development Goal target of halting TB by 2015 and beginning to reverse the incidence of TB [47].

## Additional files

## Electronic supplementary material

Additional file 1: NICE checklists. (PDF 2 MB)

Additional file 2: Comparison of ISTC and TB care practices. (DOC 60 KB)

Below are the links to the authors’ original submitted files for images.

Authors’ original file for figure 1

## Abbreviations

AFB: Acid-fast bacillus
ARDS: Acute respiratory distress syndrome
DOT: Directly observed treatment
DR: Drug resistant
HIV: Human immunodeficiency virus
IGRA: Interferon gamma release assay
ISTC: International standards for tuberculosis care
LTBI: Latent tuberculosis infection
MDR-TB: Multidrug resistant tuberculosis
PTB: Pulmonary tuberculosis
TB: Tuberculosis
TST: Tuberculin sensitivity test
WHO: World health organization
XDR-TB: Extensively drug resistant tuberculosis
