# Supplementary material for: Tuberculosis care for pregnant women: a systematic review
Source: BMC Infect Dis. 2014 Nov 19;14:617. doi: 10.1186/s12879-014-0617-x (PMC4241224; doi:10.1186/s12879-014-0617-x)
Supplement: Supplementary file 1 — Additional file 1: NICE checklists. (PDF 2 MB) [file 12879_2014_617_MOESM1_ESM.pdf]

SECTION 3: DESCRIPTION OF THE STUDY (The following information is required to complete evidence tables facilitating cross-study comparisons. Please complete all sections for which information is available) *PLEASE PRINT CLEARLY*		
3.1	How many individuals are included in this study?<br>*Please indicate number in each arm of the study, at the time the study began.*	
3.2	What are the main characteristics of the population?<br>*(Include all relevant characteristics – for example, age, sex, ethnic origin, comorbidity, disease status, community/hospital based)*	
3.3	What intervention (treatment, procedure) is being investigated in this study?<br>*List all interventions covered by the study.*	
3.4	What comparisons are made in the study?<br>*Are comparisons made between interventions, or between intervention and placebo/no intervention?*	
3.5	How long are individuals followed up in the study?<br>*Length of time individuals are followed from beginning participation in the study. Note specified end point used to decide end of follow-up (for*	

	example, death, complete cure). Note if follow-up period is shorter than originally planned.	
3.6	What outcome measure(s) are used in the study?<br>*List all outcomes that are used to assess effectiveness of the interventions used.*	
3.7	What size of effect is identified in the study?<br>*List all measures of effect in the units used in the study – for example, absolute or relative risk, number needed to treat. Include p values and any confidence intervals that are provided.*	
3.8	How was this study funded?<br>*List all sources of funding quoted in the article, whether Government, voluntary sector or industry.*	
3.9	Does this study help to answer your key question?<br>*Summarise the main conclusions of the study and indicate how it relates to the key question.*	

A.2 Notes on the use of methodology checklist: randomised controlled trials

Section 1 identifies the study and asks a series of questions aimed at establishing the internal validity of the study under review – that is, making sure that it has been carried out carefully, and that the outcomes are likely to be attributable to the intervention being investigated. Each question covers an aspect of methodology that research has shown makes a significant difference to the conclusions of a study.

For each question in this section you should use one of the following to indicate how well it has been addressed in the study.

- Well covered.
- Adequately addressed.
- Poorly addressed.
- Not addressed (that is, not mentioned, or indicates that this aspect of study design was ignored).
- Not reported (that is, mentioned, but insufficient detail to allow assessment to be made).
- Not applicable.

A.2.1 The study addresses an appropriate and clearly focused question

Unless a clear and well-defined question is specified, it will be difficult to assess how well the study has met its objectives or how relevant it is to the question you are trying to answer on the basis of its conclusions.

A.2.2 The assignment of subjects to intervention groups is randomised

Random allocation of individuals to receive one or other of the interventions under investigation, or to receive either intervention or placebo, is fundamental to this type of study. **If there is no indication of randomisation, the study should be rejected.** If the description of randomisation is poor, or the process used is not truly random (for example, allocation by date, alternating between one group and another) or can otherwise be seen as flawed, the study should be given a lower quality rating.

A.2.3 An adequate concealment method is used

Research has shown that where allocation concealment is inadequate, investigators can overestimate the effect of interventions by up to 40%. Centralised allocation, computerised allocation systems or the use of coded identical containers would all be regarded as adequate methods of concealment, and may be taken as indicators of a well-conducted study. If the method of concealment used is regarded as poor, or relatively easy to subvert, the study must be given a lower quality rating, and can be rejected if the concealment method is seen as inadequate.

A.2.4 Subjects and investigators are kept 'blind' about intervention allocation

Blinding can be carried out up to three levels. In single-blind studies, individuals are unaware of which intervention they are receiving; in double-blind studies the doctor and the individual are unaware of which intervention the individual is receiving; in triple-blind studies individuals, healthcare providers and those conducting the analysis are unaware of which individuals received which intervention. The higher the level of blinding, the lower the risk of bias in the study.

A.2.5 The intervention and control groups are similar at the start of the trial

Individuals selected for inclusion in a trial should be as similar as possible, in order to eliminate any possible bias. The study should report any significant differences in the composition of the study groups in relation to gender mix, age, stage of disease (if appropriate), social background, ethnic origin or comorbid conditions. These factors may be covered by inclusion and exclusion criteria, rather than being reported directly. Failure to address this question, or the use of inappropriate groups, should lead to the study being downgraded.

A.2.6 The only difference between groups is the intervention under investigation

If some individuals received additional treatment, even if of a minor nature or consisting of advice and counselling rather than a physical intervention, this treatment is a potential confounding factor that may invalidate the results. **If groups were *not* treated equally, the study should be rejected unless no other evidence is available.** If the study *is* used as evidence it should be treated with caution, and given a low quality rating.

A.2.7 All relevant outcomes measured in a standard, valid and reliable way

If some significant clinical outcomes have been ignored, or not adequately taken into account, the study should be downgraded. It should also be downgraded if the measures used are regarded as being doubtful in any way, or applied inconsistently.

A.2.8 What percentage of the individuals or clusters recruited into each intervention arm of the study dropped out before the study was completed?

The number of individuals that drop out of a study should give concern if the number is very high. Conventionally, a 20% drop-out rate is regarded as acceptable, but this may vary. Some regard should be paid to *why* individuals dropped out, as well as how many. It should be noted that the drop-out rate

may be expected to be higher in studies conducted over a long period of time. A higher drop-out rate will normally lead to downgrading, rather than rejection of a study.

A.2.9 All the subjects are analysed in the groups to which they were randomly allocated (often referred to as intention-to-treat analysis)

In practice, it is rarely the case that all individuals allocated to the intervention group receive the intervention throughout the trial, or that all those in the comparison group do not. Individuals may refuse treatment, or contra-indications arise that lead them to be switched to the other group. If the comparability of groups through randomisation is to be maintained, however, individual outcomes *must* be analysed according to the group to which they were originally allocated, irrespective of the intervention they actually received. (This is known as *intention-to-treat* analysis.) If it is clear that analysis was not on an intention-to-treat basis, the quality of the study should be downgraded.

A.2.10 Where the study is carried out at more then one site, results are comparable for all sites

In multi-site studies, confidence in the results should be increased if it can be shown that similar results were obtained at the different participating centres.
Section 2 relates to the overall assessment of the paper. It starts by rating the methodological quality of the study, based on your responses in Section 1 and using the following coding system:

++	All or most of the criteria have been fulfilled.<br>Where they have not been fulfilled the conclusions of the study or review are thought **very unlikely** to alter.
+	Some of the criteria have been fulfilled.<br>Those criteria that have not been fulfilled or not adequately described are thought **unlikely** to alter the conclusions.
–	Few or no criteria fulfilled.<br>The conclusions of the study are thought **likely or very likely** to alter.

The code allocated here, coupled with the study type, will decide the **level of evidence** that this study provides.
The aim of the other two questions in this section is to summarise your view of the quality of this study and its applicability to the patient group targeted by the guideline you are working on.
Section 3 asks you to summarise key points about the study that will be added to an evidence table at the next stage of the process.

A.3 Methodology checklist: case–control studies

**Study identification**<br>*Include author, title, reference, year of publication*		
**Guideline topic**		**Key question no:**
**Checklist completed by:**		
**SECTION 1: INTERNAL VALIDITY**		
**In a well conducted case–control study:**		**In this study the criterion is: (*Circle one option for each question)***
1.1	The study addresses an appropriate and clearly focused question.	Well covered / Not addressed<br>Adequately addressed / Not reported<br>Poorly addressed / Not applicable
SELECTION OF SUBJECTS		
1.2	The cases and controls are taken from comparable populations.	Well covered / Not addressed<br>Adequately addressed / Not reported<br>Poorly addressed / Not applicable
1.3	The same exclusion criteria are used for both cases and controls.	Well covered / Not addressed<br>Adequately addressed / Not reported<br>Poorly addressed / Not applicable
1.4	What percentage of each group (cases and controls) participated in the study?	Cases:<br>Controls:
1.5	Comparison is made between participants and non-participants to establish their similarities or differences.	Well covered / Not addressed<br>Adequately addressed / Not reported<br>Poorly addressed / Not applicable
1.6	Cases are clearly defined and differentiated from controls.	Well covered / Not addressed<br>Adequately addressed / Not reported<br>Poorly addressed / Not applicable
1.7	Is it clearly established that controls are non-cases?	Well covered / Not addressed<br>Adequately addressed / Not reported<br>Poorly addressed / Not applicable

**ASSESSMENT**		
1.8	Measures have been taken to prevent knowledge of primary exposure influencing case ascertainment.	Well covered / Adequately addressed / Poorly addressed / Not addressed / Not reported / Not applicable
1.9	Exposure status is measured in a standard, valid and reliable way.	Well covered / Adequately addressed / Poorly addressed / Not addressed / Not reported / Not applicable
**CONFOUNDING**		
1.10	The main potential confounders are identified and taken into account in the design and analysis.	Well covered / Adequately addressed / Poorly addressed / Not addressed / Not reported / Not applicable
**STATISTICAL ANALYSIS**		
1.11	Have confidence intervals been provided?	
**SECTION 2: OVERALL ASSESSMENT OF THE STUDY**		
2.1	How well was the study done to minimise the risk of bias or confounding?<br>*Code ++, + or –*	
2.2	Taking into account clinical considerations, your evaluation of the methodology used and the statistical power of the study, are you certain that the overall effect is due to the exposure being investigated?	
2.3	Are the results of this study directly applicable to the patient group targeted by this guideline?	

SECTION 3: DESCRIPTION OF THE STUDY (Note: The following information is required for evidence tables to facilitate cross-study comparisons. Please complete all sections for which information is available)<br>*PLEASE PRINT CLEARLY*		
3.1	How many individuals participated in the study?<br>*List the number of cases and controls separately.*	
3.2	What are the main characteristics of the study population?<br>*Include all characteristics used to identify both cases and controls – for example, age, sex, social class, disease status.*	
3.3	What environmental or prognostic factor is being investigated in this study?	
3.4	What comparisons are made in the study?<br>*Normally only one factor will be compared, but in some cases the extent of exposure may be stratified – for example, non-smokers v. light, moderate or heavy smokers. Note all comparisons here.*	
3.5	For how long are individuals followed up in the study?<br>*Length of time participant histories are tracked in the study.*	
3.6	What outcome measure is used in the study?<br>*List all outcomes that are used to assess*	

	the impact of the chosen environmental or prognostic factor.	
3.7	What size of effect is identified in the study?<br>*Effect size should be expressed as an odds ratio. If any other measures are included, note them as well. Include p values and any confidence intervals that are provided.*	
3.8	How was this study funded?<br>*List all sources of funding quoted in the article, whether Government, voluntary sector or industry.*	
3.9	Does this study help to answer your key question?<br>*Summarise the main conclusions of the study and indicate how it relates to the key question.*	

A.3 Notes on the use of methodology checklist: case-control studies

The studies covered by this checklist are designed to answer questions of the type 'What are the factors that caused this event?', and involve comparison of individuals with an outcome with other individuals from the same population who do not have the outcome. These studies start after the outcome of an event, and can be used to assess multiple causes of a single event. They are generally used to assess the causes of a new problem, but they may also be useful for the evaluation of population-based interventions such as screening.

Section 1 identifies the study and asks a series of questions aimed at establishing the internal validity of the study under review – that is, making sure that it has been carried out carefully, and that the any link between events and outcomes is clearly established. Each question covers an aspect of methodology that has been shown to make a significant difference to the conclusions of a study.

Case-control studies need to be very carefully designed, and the complexity of their design is often not appreciated by investigators, leading to many poor quality studies being conducted. The questions in this checklist are designed to identify the main features that should be present in a well-designed study. There are few criteria that should, alone and unsupported, lead to rejection of a study. However, a study that fails to address or report on more than one or two of the questions addressed below should almost certainly be rejected.

For each question in this section you should use one of the following to indicate how well it has been addressed in the study.

- Well covered.
- Adequately addressed.
- Poorly addressed.
- Not addressed
  (that is, not mentioned, or indicates that this aspect of study design was ignored).
- Not reported
  (that is, mentioned, but insufficient detail to allow assessment to be made).
- Not applicable.

A.3.1 The study addresses an appropriate and clearly focused question

Unless a clear and well-defined question is specified, it will be difficult to assess how well the study has met its objectives or how relevant it is to the question you are trying to answer on the basis of its conclusions.

A.3.2 The cases and controls are taken from comparable populations

Study participants may be selected from the target population (all individuals to which the results of the study could be applied), the source population (a defined subset of the target population from which participants are selected)

or from a pool of eligible subjects (a clearly defined and counted group selected from the source population. **If the study does not include clear definitions of the source population it should be rejected.**

A.3.3 The same exclusion criteria are used for both cases and controls

All selection and exclusion criteria should be applied equally to cases and controls. Failure to do so may introduce a significant degree of bias into the results of the study.

A.3.4 What percentage of each group (cases and controls) participated in the study?

Differences between the eligible population and the participants are important, as they may influence the validity of the study. A *participation rate* can be calculated by dividing the number of study participants by the number of eligible subjects. It is more useful if calculated separately for cases and controls. If the participation rate is low, or there is a large difference between the two groups, the study results may well be invalid due to differences between participants and non-participants. In these circumstances, the study should be downgraded, and rejected if the differences are very large.

A.3.5 Comparison is made between participants and non-participants to establish their similarities or differences

Even if participation rates are comparable and acceptable, it is still possible that the participants selected to act as cases or controls may differ from other members of the source population in some significant way. A well-conducted case–control study will look at samples of the non-participants among the source population to ensure that the participants are a truly representative sample.

A.3.6 Cases are clearly defined and differentiated from controls

The method of selection of cases is of critical importance to the validity of the study. Investigators have to be certain that cases are truly cases, but must balance this with the need to ensure that the cases admitted into the study are representative of the eligible population. **The issues involved in case selection are complex, and should ideally be evaluated by someone with a good understanding of the design of case–control studies.** If the study does not comment on how cases were selected, it is probably safest to reject it as a source of evidence.

A.3.7 Is it clearly established that controls are non-cases?

Just as it is important to be sure that cases are true cases, it is important to be sure that controls do not have the outcome under investigation. Control subjects should be chosen so that information on exposure status can be obtained or assessed in a similar way to that used for the selection of cases. If the methods of control selection are not described, the study should be rejected. **If different methods of selection are used for cases and controls**

the study should be evaluated by someone with a good understanding of the design of case–control studies.

A.3.8 Measures will have been taken to prevent knowledge of primary exposure influencing case ascertainment

If there is a possibility that case ascertainment can be influenced by knowledge of exposure status, assessment of any association is likely to be biased. A well-conducted study should take this into account in the design of the study.

A.3.9 Exposure status is measured in a standard, valid and reliable way

The inclusion of evidence from other sources or previous studies that demonstrate the validity and reliability of the assessment methods used, or that the measurement method used is a recognised procedure, should further increase confidence in study quality.

A.3.10 The main potential confounders are identified and taken into account in the design and analysis

Confounding is the distortion of a link between exposure and outcome by another factor that is associated with both exposure and outcome. The possible presence of confounding factors is one of the principal reasons why observational studies are not more highly rated as a source of evidence. The report of the study should indicate which potential confounders have been considered, and how they have been assessed or allowed for in the analysis. Clinical judgement should be applied to consider whether all likely confounders have been considered. If the measures used to address confounding are considered inadequate, the study should be downgraded or rejected, depending on how serious the risk of confounding is considered to be. **A study that does not address the possibility of confounding should be rejected.**

A.3.11 Have confidence intervals been provided?

Confidence limits are the preferred method for indicating the precision of statistical results, and can be used to differentiate between an inconclusive study and a study that shows no effect. Studies that report a single value with no assessment of precision should be treated with caution.

Section 2 relates to the overall assessment of the paper. It starts by rating the methodological quality of the study, based on your responses in Section 1 and using the following coding system:

++	**All or most** of the criteria have been fulfilled.<br>Where they have not been fulfilled the conclusions of the study or review are thought *very* **unlikely** to alter.
+	**Some** of the criteria have been fulfilled.<br>Those criteria that have not been fulfilled or not adequately described

	are thought **unlikely** to alter the conclusions.
–	**Few or no** criteria fulfilled<br>The conclusions of the study are *thought* **likely or very likely** to alter.

The code allocated here, coupled with the study type, will decide the **level of evidence** that this study provides.

The aim of the other two questions in this section is to summarise your view of the quality of this study and its applicability to the patient group targeted by the guideline you are working on.

Section 3 asks you to summarise key points about the study that will be added to an evidence table at the next stage of the process.

A.4 Methodology checklist: Qualitative studies

**Study identification**<br>*Include author, title, reference, year of publication*	
**Guideline topic:**	**Key question no:**
**Checklist completed by:**	

**Criterion**	**Is the criterion clearly addressed?**	
**Epistemology**		
**1. Is a qualitative approach appropriate?**<br>• Does the research seek to understand processes or structures, or illuminate subjective experiences or meanings?<br>• Could a quantitative approach better have addressed the question	☐ Appropriate<br>☐ Inappropriate<br>☐ Not sure	Comments:
**2. Is the study clear in what it seeks to do?**<br>• Is the purpose of the research discussed – aims/objectives/research question<br>• Is there adequate reference to the literature<br>• Are underpinning values/assumptions/theory discussed	☐ Clear<br>☐ Unclear	Comments:

Study Design		
**3. How defensible is the research design?**<br>• Is the design appropriate to the question<br>• Are there clear accounts of the criteria used for sampling, data collection, data analysis<br>• Is the selection of cases/sampling strategy theoretically justified<br>• Is a rationale given for the choice of method	☐ Defensible<br>☐ Indefensible<br>☐ Not sure	Comments:

Data collection		
**4. How well was the data collection carried out?**<br>• Were the data collected in a way which addressed the research question<br>• Was the data collection and record keeping systematic	☐ Appropriately<br>☐ Inappropriately<br>☐ Not sure	Comments:

Validity		
**5. Is the role of the researcher clearly described?**<br>• Has the relationship between the researcher and the participants been adequately considered<br>• Is there evidence about how the research was explained and presented to the participants	☐ Clear<br>☐ Unclear<br>☐ Not sure	Comments:
**6. Is the context clearly described?**<br>• Are the characteristics of the participants and settings clearly defined<br>• Were observations made in a sufficient variety of circumstances<br>• Was context bias considered	☐ Clear<br>☐ Unclear<br>☐ Not sure	Comments:
**7. Were the methods reliable?**<br>• Was data collected by more than one method<br>• Is there triangulation, or justification for not triangulating<br>• Do the methods investigate what they claim to	☐ Reliable<br>☐ Unreliable<br>☐ Not sure	Comments:

Analysis		
**8. Is the data analysis sufficiently rigorous?**<br><br>• Is the procedure explicit – i.e. is it clear how the data was processed to arrive at the results<br>• How systematic is the analysis, is the procedure reliable/dependable<br>• Is it clear how the themes and concepts were derived from the data	☐ Rigorous<br><br>☐ Not rigorous<br><br>☐ Not sure	Comments:
**9. Is the data rich?**<br><br>• How well are the contexts of the data preserved<br>• Has the diversity of perspective and content been explored<br>• How well has the detail and depth been preserved<br>• Are responses compared and contrasted across groups/sites	☐ Rich<br><br>☐ Poor<br><br>☐ Not sure	Comments:
**10. Is the analysis reliable?**<br><br>• Did more than one researcher theme and code transcripts<br>• If so, how were differences resolved<br>• Did participants feed back on the data if possible and relevant<br>• Were negative/ discrepant results addressed or ignored	☐ Reliable<br><br>☐ Unreliable<br><br>☐ Not sure	Comments:
**11. Are the findings credible?**<br><br>• Is there a clear statement of the findings	☐ Credible<br><br>☐ Not credible	Comments:

• Are the findings internally coherent<br>• Are elements from the original data included<br>• Can the data sources be traced<br>• Is the reporting clear and coherent	☐ Not sure	
**12. Are the findings relevant?**	☐ Relevant<br>☐ Irrelevant<br>☐ Not sure	Comments:
**13. Conclusions**<br>• How clear are the links between data, interpretation and conclusions<br>• Are the conclusions plausible and coherent<br>• Have alternative explanations been explored and discounted<br>• Does this enhance understanding of the research topic<br>• Are the implications clearly defined<br>• Is there adequate discussion of limitations	☐ Adequate<br>☐ Inadequate<br>☐ Not sure	Comments:

**Ethics**		
**14. How clear and coherent is the reporting of ethics?**<br>• Have ethical issues been taken into consideration<br>• Are they adequately discussed e.g. do they address consent and anonymity<br>• Have the consequences of the research been considered i.e. raising expectations, changing behaviour etc<br>• Was the study approved by an ethics committee	☐ Appropriate<br>☐ Inappropriate<br>☐ Not sure	Comments:
**Overall Assessment**		
**Is this study relevant?**	☐Yes ☐No	Comments:
**How well was the study conducted? (see table below)**	☐ ++<br>☐ +<br>☐ -	

++	All or most of the criteria have been fulfilled.<br>Where they have not been fulfilled the conclusions of the study or review are thought **very unlikely** to alter.
+	Some of the criteria have been fulfilled.<br>Those criteria that have not been fulfilled or not adequately described are thought **unlikely** to alter the conclusions.
–	Few or no criteria fulfilled.<br>The conclusions of the study are thought **likely or very likely** to alter.

A.5 Methodology checklist: Cohort studies (adapted from Tooth et al. 2005)*

**Study identification**<br>*Include author, title, reference, year of publication*		
**Guideline topic:**	**Key question no:**	
**Checklist completed by:**		
1. Are the objectives or hypotheses of the study stated?	Well covered<br>Adequately addressed<br>Poorly addressed	Not addressed<br>Not reported<br>Not applicable
2. Is the target population defined?	Well covered<br>Adequately addressed<br>Poorly addressed	Not addressed<br>Not reported<br>Not applicable
3. Is the sampling frame defined?	Well covered<br>Adequately addressed<br>Poorly addressed	Not addressed<br>Not reported<br>Not applicable
4. Is the study population defined?	Well covered<br>Adequately addressed<br>Poorly addressed	Not addressed<br>Not reported<br>Not applicable
5. Are the study setting (venues) and/or geographic location stated?	Well covered<br>Adequately addressed<br>Poorly addressed	Not addressed<br>Not reported<br>Not applicable
6. Are the dates between which the study was conducted stated or implicit?	Well covered<br>Adequately addressed<br>Poorly addressed	Not addressed<br>Not reported<br>Not applicable
7. Are eligibility criteria stated?	Well covered<br>Adequately addressed<br>Poorly addressed	Not addressed<br>Not reported<br>Not applicable
8. Are issues of 'selection in' to the study mentioned? **	Well covered<br>Adequately	Not addressed

	addressed<br>Poorly addressed	Not reported<br>Not applicable
9. Is the numbers of participants justified?	Well covered<br>Adequately addressed<br>Poorly addressed	Not addressed<br>Not reported<br>Not applicable
10. Are the numbers meeting and not meeting the eligibility criteria stated?	Well covered<br>Adequately addressed<br>Poorly addressed	Not addressed<br>Not reported<br>Not applicable
11. For those not eligible, are the reasons why stated?	Well covered<br>Adequately addressed<br>Poorly addressed	Not addressed<br>Not reported<br>Not applicable
12. Are the numbers of people who did/did not consent to participate stated?	Well covered<br>Adequately addressed<br>Poorly addressed	Not addressed<br>Not reported<br>Not applicable
13. Are the reasons that people refused to consent stated?	Well covered<br>Adequately addressed<br>Poorly addressed	Not addressed<br>Not reported<br>Not applicable
14. Were consenters compared with nonconsenters?	Well covered<br>Adequately addressed<br>Poorly addressed	Not addressed<br>Not reported<br>Not applicable
15. Was the number of participants at the beginning of the study stated?	Well covered<br>Adequately addressed<br>Poorly addressed	Not addressed<br>Not reported<br>Not applicable
16. Were the methods of data collection stated?	Well covered<br>Adequately addressed<br>Poorly addressed	Not addressed<br>Not reported<br>Not applicable
17. Was the reliability (repeatability) of measurement methods mentioned?	Well covered<br>Adequately addressed<br>Poorly addressed	Not addressed<br>Not reported<br>Not applicable

18. Was the validity (against a "gold standard") of measurement methods mentioned?	Well covered<br>Adequately addressed<br>Poorly addressed	Not addressed<br>Not reported<br>Not applicable
19. Were any confounders mentioned?	Well covered<br>Adequately addressed<br>Poorly addressed	Not addressed<br>Not reported<br>Not applicable
20. Was the number of participants at each stage/wave specified?	Well covered<br>Adequately addressed<br>Poorly addressed	Not addressed<br>Not reported<br>Not applicable
21. Were reasons for loss to follow-up quantified?	Well covered<br>Adequately addressed<br>Poorly addressed	Not addressed<br>Not reported<br>Not applicable
22. Was the missingness of data items at each wave mentioned?	Well covered<br>Adequately addressed<br>Poorly addressed	Not addressed<br>Not reported<br>Not applicable
23. Was the type of analyses conducted stated?	Well covered<br>Adequately addressed<br>Poorly addressed	Not addressed<br>Not reported<br>Not applicable
24. Were "longitudinal" analysis methods stated?	Well covered<br>Adequately addressed<br>Poorly addressed	Not addressed<br>Not reported<br>Not applicable
25. Were absolute effect sizes reported?	Well covered<br>Adequately addressed<br>Poorly addressed	Not addressed<br>Not reported<br>Not applicable
26. Were relative effect sizes reported?	Well covered<br>Adequately addressed<br>Poorly addressed	Not addressed<br>Not reported<br>Not applicable
27. Was loss to follow-up taken into account in the analysis?	Well covered<br>Adequately	Not addressed<br>Not reported

	addressed<br>Poorly addressed	Not applicable
28. Were confounders accounted for in analyses?	Well covered<br>Adequately addressed<br>Poorly addressed	Not addressed<br>Not reported<br>Not applicable
29. Were missing data accounted for in the analyses?	Well covered<br>Adequately addressed<br>Poorly addressed	Not addressed<br>Not reported<br>Not applicable
30. Was the impact of biases assessed qualitatively?	Well covered<br>Adequately addressed<br>Poorly addressed	Not addressed<br>Not reported<br>Not applicable
31. Was the impact of biases estimated quantitatively?	Well covered<br>Adequately addressed<br>Poorly addressed	Not addressed<br>Not reported<br>Not applicable
32. Did authors related results back to a target population?	Well covered<br>Adequately addressed<br>Poorly addressed	Not addressed<br>Not reported<br>Not applicable
33. Was there any other discussion of generalisability?	Well covered<br>Adequately addressed<br>Poorly addressed	Not addressed<br>Not reported<br>Not applicable
34. **Overall Assessment of Study.**<br>How well was the study done to minimise the risk of bias or confounding, and to establish a causal relationship between exposure and effect?<br>Code ++, + or -		

*Criteria taken from: Tooth L, Ware R, Bain C et al. (2005) Quality of reporting of observational longitudinal research. *American Journal of Epidemiology* vol 161(3):280-288.

**Represents selection bias at the beginning of study. Other biases (i.e. loss to follow-up, missing data items) are dealt with by other checklist criteria

A.5 Notes on the use of methodology checklist: cohort studies

Criteria taken from: Tooth L et al. (2005) Quality of reporting of observational longitudinal research. *American Journal of Epidemiology* 161(3):280-288.

1. Self-explanatory
2. The group of persons toward whom inferences are directed. Sometimes the population from which a study group is drawn
3. The list of units from which the study population will be drawn. Ideally, the sampling frame would be identical to the target population, but it is not always possible
4. The group selected for investigation
5. Comment required about location of research. Could include name of centre, town, or district
6. Self-explanatory
7. The words 'eligibility criteria' or equivalent are needed, unless the entire population is the study population
8. Any aspect of recruitment or setting that results in the selective choice of participants (e.g. gender or health status influenced recruitment)
9. Justification of number of subjects needed to detect anticipated effects. Evidence that power calculations were considered and/or conducted
10. Quantitative statement of numbers
11. Broad mention of the major reasons
12. Quantitative statement of numbers
13. Broad mention of the major reasons
14. Quantitative comparison of the different groups
15. Total number of participants (after screening for eligibility and consent) included in the first stage of data collection
16. Descriptions of tools (e.g. Surveys, physical examinations) and processes (e.g. Face-to-face, telephone)
17. Evidence of reproducibility of the tools used
18. Evidence that the validity was examined against, or discussed in relation to, a gold standard
19. Confounders were defined as a variable that can cause or prevent the outcome of interest, is not an intermediate variable, and is associated with the factors under investigation
20. Quantitative statement of numbers at each follow-up point
21. Broad mention and quantification of the major reasons
22. Differences in numbers of data points (indicating missing data items) explained
23. Specific statistical methods mentioned by name
24. Longitudinal analyses were defined as those assessing change in outcome over two or more time points and that take into account the fact that the observations are likely to be correlated
25. Absolute effect was defined as the outcome of an exposure expressed, for example, as the difference between rates, proportions, or means, as opposed to the ratios of these measures
26. Relative effects were defined as a ratio of rates, proportions, or other measure of an effect
27. Specific mention of adjusting for, or stratifying by, loss to follow-up

28. Specific mention of adjusting for, or stratifying by, confounders
29. Specific mention of adjusting for, or stratifying by, or imputation of missing data items
30. Specific mention of bias affecting results, but magnitude not quantified
31. Specific mention of numerical magnitude of bias
32. A study is generalisable if it can produce unbiased inferences regarding a target population (beyond the subjects in the study). Discussion could include that generalisability is not possible
33. Discussion of generalisability beyond the target population

A.6 Methodology checklist: Controlled Before and After studies (adapted from 'EPOC' by Cardiff University PHCC)

**Study identification**<br>*Include author, title, reference, year of publication*	
**Guideline topic:**	**Key question no:**
**Checklist completed by:**	

This checklist to be used for studies where there is involvement of intervention and control groups other than by random process, and inclusion of baseline period of assessment of main outcomes. There are two minimum criteria for inclusion of CBAs in EPOC reviews:

a) ***Contemporaneous data collection***

- Score DONE pre and post intervention periods for study and control sites are the same.
- Score NOT CLEAR if it is not clear in the paper, e.g. dates of collection are not mentioned in the text. (N.B. the paper should be discussed with the contact editor for the review before data extraction is undertaken).
- Score NOT DONE if data collection was not conducted contemporaneously during pre and post intervention periods for study and control sites.

b) ***Appropriate choice of control site***

- Studies using second site as controls:
- Score DONE if study and control sites are comparable with respect to dominant rèimbursement system, level of care, setting of care and academic status.
- Score NOT CLEAR if not clear from paper whether study and control sites are comparable. (N.B. the paper should be discussed with the contact editor for the review before data extraction is undertaken).
- Score NOT DONE if study and control sites are not comparable.

Quality criteria for controlled before and after (CBA) designs

Seven standard criteria are used for CBAs included in EPOC reviews:

a) **Baseline measurement**

- Score DONE if performance or patient outcomes were measured prior to the intervention, and no substantial differences were present across study groups (e.g. where multiple pre intervention measures describe similar trends in intervention and control groups);
- Score NOT CLEAR if baseline measures are not reported, or if it is unclear whether baseline measures are substantially different across study groups;
- Score NOT DONE if there are differences at baseline in main outcome measures likely to undermine the post intervention differences (e.g. are differences between the groups before the intervention similar to those found post intervention).

b) **Characteristics for <u>studies using second site as control</u>**

- Score DONE if characteristics of study and control providers are reported and similar;
- Score NOT CLEAR if it is not clear in the paper e.g. characteristics are mentioned in the text but no data are presented;
- Score NOT DONE if there is no report of characteristics either in the text or a table OR if baseline characteristics are reported and there are differences between study and control providers.

c) **Blinded assessment of primary outcome(s)* (protection against detection bias)**

- Score DONE if the authors state explicitly that the primary outcome variables were assessed blindly OR the outcome variables are objective e.g. length of hospital stay, drug levels as assessed by a standardised test;
- Score NOT CLEAR if not specified in the paper;
- Score NOT DONE if the outcomes were not assessed blindly.

** Primary outcome(s) are those variables that correspond to the primary hypothesis or question as defined by the authors. In the event that some of the primary outcome variables were assessed in a blind fashion and others were not, score each separately and label each outcome variable clearly.*

d) **Protection against contamination**

- Studies using second site as control
- Score DONE if allocation was by community, institution, or practice and is unlikely that the control group received the intervention;
- Score NOT CLEAR if providers were allocated within a clinic or practice and communication between experimental and group providers was likely to occur;
- Score NOT DONE if it is likely that the control group received the intervention (e.g. cross-over studies or if individuals rather than providers were randomised).

e) **Reliable primary outcome measure(s)**

- Score DONE if two or more raters with at least 90% agreement or kappa greater than or equal to 0.8 OR the outcome is obtained from some automated system e.g. length of hospital stay, drug levels as assessed by a standardised test;
- Score NOT CLEAR if reliability is not reported for outcome measures that are obtained by chart extraction or collected by an individual;
- Score NOT DONE if agreement is less than 90% or kappa is less than 0.8.

** In the event that some outcome variables were assessed in a reliable fashion and others were not, score each separately and label each outcome variable clearly.*

f) **Follow-up of professionals (protection against exclusion bias)**

- Score DONE if outcome measures obtained 80-100% subjects allocated to groups. (Do not assume 100% follow-up unless stated explicitly.);
- Score NOT CLEAR if not specified in the paper;
- Score NOT DONE if outcome measures obtained for less than 80% of individuals allocated to groups.

g) **Follow-up of individuals**

- Score DONE if outcome measures obtained 80-100% of individuals allocated to groups or for individuals who entered the study. (Do not assume 100% follow-up unless stated explicitly.);
- Score NOT CLEAR if not specified in the paper;

- Score NOT DONE if outcome measures obtained for less than 80% of individuals allocated to groups or for less than 80% of individuals who entered the study.

**OVERALL ASSESSMENT OF STUDY**	
How well was the study conducted? Code ++, + or –	
Are the results of the study directly applicable to the groups targeted by the guideline?	
