# Supplementary material for: Tuberculosis care for pregnant women: a systematic review
Source: BMC Infect Dis. 2014 Nov 19;14:617. doi: 10.1186/s12879-014-0617-x (PMC4241224; doi:10.1186/s12879-014-0617-x)
Supplement: Supplementary file 2 — Additional file 2: Comparison of ISTC and TB care practices. (DOC 60 KB) [file 12879_2014_617_MOESM2_ESM.doc]

**Additional file 1.** Comparison of ISTC and TB care practices

| International standards for TB care* | | No. patients in reviewed studies whose care corresponded with ISTC/total patients of group defined in ISTC ** | Notes |
| --- | --- | --- | --- |
| **Diagnosis/screening** | | | |
| 1. All persons with other wise unexplained productive cough lasting 2-3 weeks or more should be evaluated for TB | 285/285 | | In 4878 pregnant TB patients, only 285 had this symptom |
| 1. All patients suspected of having PTB who are capable of producing sputum should have at least 2, and preferably 3, sputum specimens obtained for microscopic examination. When possible at least one early morning specimen should be obtained | 274/320 | | In reviewed studies that mentioned sputum specimen collection, sputum specimens were only collected 2 times |
| 1. In all patients suspected of having extra pulmonary TB, appropriate specimens from the suspected sites of involvement should be obtained for microscopy and, where facilities and resources are available, for culture and histopathological examination | 65/95 collected specimens for microscopy; 47/95 collected specimens for culture | | In resource-poor countries, especially India, TB culture is not common practice due to unavailability of facilities in health institutions, while TB culture was conducted in all related reviewed studies from resource-rich countries |
| 1. All persons with chest radiographic findings suggestive of TB should have sputum specimen submitted for microbiological examination | 36/36 | | Chest X-ray is a diagnostic test and in pregnant women is generally taken after microbiological examination. This number is only counted on patients having chest X-ray diagnosis before sputum collection |
| 1. The diagnosis of sputum smear negative PTB should be based on the following criteria: at least 3 negative sputum smears (at least one early morning specimen); chest X-ray finding consistent with TB; and lack of response to a trial of broad-spectrum antimicrobial agents (fluoroquinolones should be avoided because they may be active with TB). For such patients if facilities for culture are available, sputum culture should be obtained. In persons with known or suspected HIV infection, the diagnostic evaluation should be expedited | 33/137 had signs of TB with chest X-ray, 13/137 were diagnosed via clinical symptoms and empirical treatment, 87/137 were diagnosed by positive culture | | In reviewed articles, HIV positive patients were diagnosed with similar procedures as non-HIV patients |
| Treatment | | | |
| 1. The practitioner must not only prescribe an appropriate regimen but should also be capable of assessing the patient’s adherence to the regimen and addressing poor adherence when it occurs | 689/689 | | All reviewed studies recorded patients’ treatment compliance (until they finished the regimen, the study ended, they delivered, or they dropped out of treatment). Higher incompliance recorded in women undergoing LTBI prophylaxis |
| 1. All patients (including those with HIV infection) who have not been treated previously should receive an internationally accepted, first-line treatment regimen using drugs of known bioavailability. The initial phase should consist of two months of INH and RIF given for 4 months. INH and EMB given for 6 months is an alternative continuation phase regime that may be used when adherence cannot be assessed but is associated with a higher rate of failure and relapse, especially in patients with HIV infection. The doses of anti-TB drugs used should conform to international recommendations. Fixed-dose combinations of 2 (INH and RIF), 3 (INH, RIF, and PZA), and 4 (INH, RIF, PZA, and EMB) drugs are highly recommended, especially when medication ingestion is not observed | | 2HREZ/7HE | 2/177 | | --- | --- | | 2HREZ/7HER | 1/177 | | 2HREZ/4HR | 5/177 | | 2HRE/5HE | 1/177 | | 9HRE | 3/177 | | HRE(unclear duration) | 10/177 | | 2HRE/7HE | 18/177 | | 2HRZ/7HR | 6/177 | | HRZ (unclear duration) | 4/177 | | 10HE | 2/177 | | 2RZorHE/4HR(E) | 111/177 | | HE(unclear duration) | 9/177 | | 12-18HE | 5/177 | | | TB therapy varied between studies and patients and was modified in specific cases such as in patients with renal disease, liver disease, HIV infection, and different types of extra-PTB |
| 1. Preferred treatment for persons not treated previously: INH, RIF, PZA, EMB daily for 2 months, or 3 times/week for 2 months. Continuation phase: INH, RIF daily for 4 months or 3 times/weeks for 2 months (EMB may be omitted in the initial phase if patients have a negative sputum smear, do not have extensive PTB and/or extra PTB, or are known to be HIV negative |
| Optional treatment for persons not treated previously: INH, RIF, PZA, EMB daily in 3 months then INH, EMB daily in 6 months.(EMB may be omitted in the initial phase if patients have negative sputum smear, do not have extensive PTB and/or extra PTB, and who know to be HIV negative |
| 1. Streptomycin is not recommend for pregnant women due to the risk of deafness in the babies | 342/375 | | Only 1 study has involved 33 women who had used streptomycin in their past pregnancy (before their pregnancy was diagnosed) |
| 1. To foster and assess adherence, a patient-centered approach to administration of drug treatment, based on the patient's needs and mutual respect between the patient and the provider, should be developed for all patients. Supervision and support should be gender- sensitive and age-specific and should draw on the full range of recommended interventions and available support services, including patient counseling and education. A central element of the patient-centered strategy is the use of measures to assess and promote adherence to the treatment regimen and to address poor adherence when it occurs. These measures should be tailored to the individual patient's circumstances and mutually acceptable to the patient and the provider. Such measures may include direct observation of medication ingestion (DOT) by a treatment supporter who is acceptable and accountable to the patient and to the health system | 5/50 (out-patients) with DOT, 45/50 checked the pill bottle during bi-monthly clinical visits | | DOT was only applied in out-patients. |
| 1. A written record of all medications given, bacteriologic responses, and adverse reactions should be maintained for all patients | 689/689 | | Yes, since all related articles are clinical studies |
| 1. In areas with high prevalence of HIV infection in the general population where TB and HIV infection are likely to co-exist, HIV counseling and testing is indicated for all TB patients as part of their routine management. In areas with lower prevalence rate of HIV, HIV counseling and testing is indicated for TB patients with symptoms and/or signs of HIV-related conditions and in TB patients with a history suggestive of high risk of HIV exposure | 318/323 TB patients were tested for HIV. In a study involving 141 TB patients in South Africa, the HIV test was mentioned without specific numbers involved. | | Some studies had no information on the HIV test so were excluded from this number. Counseling was rarely mentioned except in South Africa, where PMTCT program is combined with antenatal care in certain hospitals and patients received the HIV test before TB screening. |
| 1. All patients with TB and HIV infection should be evaluated to determine if antiretroviral therapy is indicated during the course of treatment for TB. Appropriate arrangements for access to antiretroviral drugs should be made for patients who meet indications for treatment. Given the complexity of co-administration of anti-TB treatment and antiretroviral therapy, consultation with a physician who is expert in this area is recommended before initiation of concurrent treatment for TB and HIV infection, regardless of which disease appear first. However, initiation of treatment for TB should not be delayed. Patients with TB and HIV infection should also receive co-trimoxazole as prophylaxis for other infections | 14/14 | | In reviewed articles that mentioned treatment for pregnant women with TB-HIV co-infection, all HIV patients had concurrent treatment including antiretroviral drugs and follow up and counseling by PMTCT program in high prevalence regions such as South Africa and India. Co-trimoxazole is mentioned but not in detail. |
| 1. An assessment of the likelihood of drug resistance, based on history of prior treatment, exposure to a possible source case having drug-resistance, should be obtained for all patients. Patients who fail treatment and chronic cases should always be assessed for possible drug resistance. For patients in whom drug resistance is considered to be likely, culture and drug susceptibility testing for INH,RFP and ETB should be performed promptly | 51/51 | | These issues were mentioned in studies on MDR, which involved DR test for patients with history of TB treatment. |
| 1. Patients with TB caused by drug-resistant (especially MDR) organisms should be treated with specialized regimens containing second-line anti-TB drugs. At least 4 drugs to which the organisms are known or presumed to be susceptible should be used, and treatment should be given for at least 18 months. Patient-centered measures are required to ensure adherence. Consultation with a provider experienced in treatment of patients with MDR TB should be obtained | 51/51 used 5 anti-TB drugs including 3-4 drugs in the 2nd line groups, 43/51 took drugs for more than 18 months | | 1 patient lost to follow-up after delivery, 3 died within 18 months, before completing treatment. |

*This is general standards with notion of avoiding streptomycin during pregnancy. Standards for research and public health responsibilities (with general communities) are excluded since they are not applied for reviewed studies

**Only count pregnant women with TB.
