# Supplementary figures and images for: Tuberculosis care for pregnant women: a systematic review
Source: BMC Infect Dis. 2014 Nov 19;14:617. doi: 10.1186/s12879-014-0617-x (PMC4241224; doi:10.1186/s12879-014-0617-x)

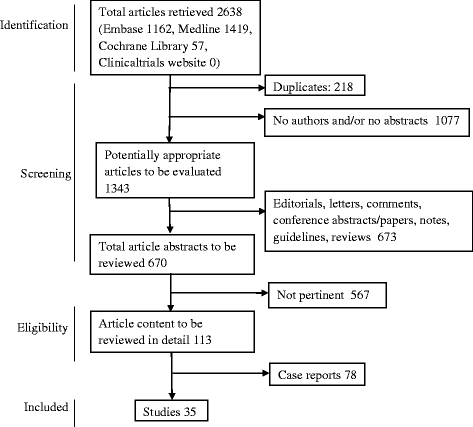

Supplement: Supplementary file 3 — Authors’ original file for figure 1 [file 12879_2014_617_MOESM3_ESM.gif]
